# An atypical case of right ventricular myxoma, a paradox of pulmonary valve endocarditis with right ventricular outflow tract obstruction. From a surgeon’s perspective

**DOI:** 10.1093/jscr/rjae592

**Published:** 2024-09-18

**Authors:** Muhammad Juffri Samsuddin, Siti Sara Yaacob, Abdul Rais Bin Sanusi

**Affiliations:** Department of Cardiovascular & Thoracic Surgery, Faculty of Medicine, Universiti Teknologi MARA, Sungai Buloh Campus, Sungai Buloh 47000, Selangor, Malaysia; Department of Public Health Medicine, Faculty of Medicine, Universiti Teknologi MARA, Jalan Hospital, Sungai Buloh Campus, Selangor Branch, Sungai Buloh 47000, Selangor, Malaysia; Department of Cardiothoracic & Vascular Surgery, National Heart Institute (IJN), No 145, Jalan Tun Razak, Kuala Lumpur 50400, Malaysia

**Keywords:** intracardiac tumours, cardiac surgery, infective endocarditis, right ventricular outflow tract obstruction, cardiac myxoma, right ventricle myxoma

## Abstract

Intracardiac tumour is rare, and intracardiac myxoma is the most common intracardiac tumour of the heart. The majority of these tumours arise at the left and right atrium, and a presentation of such a tumour other than the mentioned is atypical and very rare. Due to the rare occurrence, clinicians often misdiagnose it as infective endocarditis especially if the tumour is located near the valves and causing outflow gradient stenosis on echocardiography. A multi-modal cardiac imaging and a multi-disciplinary approach are paramount to make a correct diagnosis and treatment strategies. We would like to report a rare case of a right ventricular myxoma of a young girl, who was initially treated with infective endocarditis, which turned out to be a rare atypical Right ventricular myxoma, which was then surgically excised. The patient was successfully discharged after 3 years of follow up echocardiography showed free of tumour recurrence.

## Introduction

Intracardiac myxoma is the most common tumour of the heart, with an estimated incidence of 0.5 cases per million people per year [1]. Approximately 75% of these tumours arise from the left atrium and 18% from the right atrium. Few others originate from atypical sites such as left or right ventricles and valves [2]. The majority of myxomas (75%) are located in the left atrium whereas right ventricular myxomas are only found in 2%–4% of cases [3]. For a cardiac myxoma to present as infective endocarditis is rare [4], however, to confirm the exact diagnosis with multiple imaging modalities is paramount to give the best treatment option to the patient. We would like to report a rare presentation of a young female who was initially presented with clinical features of pulmonary valve endocarditis right ventricular outflow tract obstruction, which then underwent a successful surgical removal of a rare right ventricular myxoma with no evidence of recurrence after 3 years follow up.

## Case report

We would like to report a case of a young 18-year-old teenager, with no known co-morbidity, presented with a sudden onset of dyspnoea associated with on and off fever. There was an incidental finding of a loud systolic murmur upon initial assessment, but there were no stigmata of acute heart failure. All septic parameters were negative at that point.

**Figure 1:**
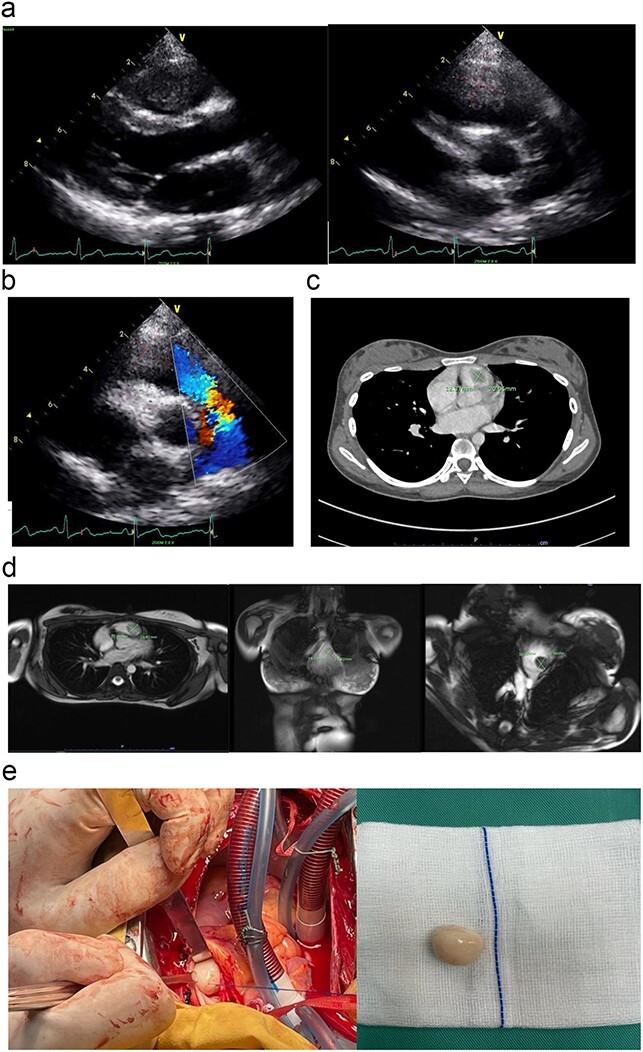
(a) The parasternal long-axis view of transthoracic ECHO showing a well-defined mass, measuring ~ (2.0 × 2.0 cm) at the right ventricle. (b) The transthoracic ECHO colour flow Doppler showed significant pulmonary valve stenosis with potential Right ventricular outflow tract obstruction caused by the intracardiac mass. (c) The axial cut of a contrast-enhanced computed tomography (CECT) thorax showed a well-defined mass measuring ~ (2 cm × 2 cm) inside the RV. (d) The cardiac magnetic resonance imaging (MRI) confirmed the diagnosis of right RV myxoma by showing a well-defined hypointense mass measuring ~ (1.7 × 2.0 cm). (e) The tumour was identified and excised via a median sternotomy, bicaval cannulation, cardiopulmonary bypass, hypothermia, and cardiac arrested via antegrade cold-blood cardioplegia. The tumour was excised via a pulmonary arteriotomy approach. The tumour is well-defined, firm in consistency, and smooth surface. The size is ~ (2 cm × 2 cm).

The initial echocardiography (ECHO) assessment findings were consistent with huge vegetation or a mass, obstructing the right ventricular outflow tract (RVOT) and abutting the pulmonary valve leaflet (Fig. 1a). She was initially treated for sterile infective endocarditis for a week duration until she was transferred to our cardiac centre for surgical intervention. After an expert opinion and multi-disciplinary consensus from the cardiologist, and surgeon, the diagnosis of right ventricular (RV) myxoma was confirmed using a multi-modality cardiac imaging such as cardiac computed tomography scan (CT scan) and cardiac magnetic resonance imaging (Fig. 1b–d). The patient was then counselled for surgical intervention.

We performed the surgical removal of RV myxoma via a primary median sternotomy. A cardiopulmonary bypass is achieved using a central aortic and bicaval cannulation. The heart was vented via the right superior pulmonary vein and an aortic root vent. The patient was then cooled down to moderate hypothermia at 30°C. An aortic cross-clamp and an antegrade cold-blood cardioplegia were given to achieve diastolic arrest. We approach the mass by performing a horizontal arteriotomy over the main pulmonary artery, to inspect the pulmonary valve leaflet and the RVOT (Fig. 1e). After the complete removal of the myxoma (Fig. 1e), the pulmonary valve was tested again before the arteriotomy closure. The patient was then rewarmed to normothermia, and the cardiopulmonary bypass was terminated. The chest was then closed in the usual manner after a post-operative transoesophageal ECHO confirmed a patent pulmonary valve and no residual mass.

The patient underwent the surgery smoothly, with no post-operative complications, and was discharged on post-operative Day 7. The post-operative ECHO showed a complete removal of the mass and a complete opening of the RVOT and a patent pulmonary valve. The histopathology report confirmed the diagnosis of a myxoma. The patient has been followed up for 3 years with yearly ECHO assessment to look for recurrence. The patient was then discharged to the district hospital after 3 years of no recurrence and free from surgical complications.

## Discussion

Intracardiac myxoma is the most common tumour of the heart, with an estimated incidence of 0.5 cases per million people per year [1]. Approximately 75% of these tumours arise from the left atrium and 18% from the right atrium. Few others originate from atypical sites such as left or right ventricles and valves [2]. The majority of myxomas (75%) are located in the left atrium whereas right ventricular myxomas are only found in 2%–4% of cases [3]. For a cardiac myxoma to present as infective endocarditis is rare [4], however, to confirm the exact diagnosis with multiple imaging modalities is paramount to give the best treatment option to the patient.

A holistic approach and a multi-disciplinary discussion amongst surgeons, cardiologists, radiologists, and anaesthesiologists are needed to confirm the pre-operative diagnosis, surgical planning, and post-operative management. Any misdiagnosis at this point may lead to tremendous effects on the patient as both the diagnosis of myxoma and infective endocarditis have different surgical and medical strategies.

Prognosis post excision is excellent, with survival rates consistently quoted at >90% with reoccurrence rates of ~5% [5]. There are no definitive guidelines, but yearly surveillance echocardiography is a reasonable strategy as early diagnosis and intervention reduce potential complications [4]. As in this case, we conducted a yearly follow-up with an ECHO assessment to look for recurrence, and patient symptoms or signs of heart failure post-operatively. The patient was discharged after 3 years follow up and in the third year ECHO showed no evidence of tumour recurrence.

## Conclusion

Right ventricular cardiac myxoma is a rare benign cardiac tumour and may initially mistaken for pulmonary valve endocarditis. A multi-modal cardiac imaging and a multi-disciplinary approach are paramount in establishing the exact diagnosis and treatment strategies. Yearly follow-up with serial echocardiography assessment is crucial to look for any tumour recurrence.
